# Spatial Topological Relation Analysis for Cluttered Scenes

**DOI:** 10.3390/s20247181

**Published:** 2020-12-15

**Authors:** Yu Fu, Mantian Li, Xinyi Zhang, Sen Zhang, Chunyu Wei, Wei Guo, Hegao Cai, Lining Sun, Pengfei Wang, Fusheng Zha

**Affiliations:** 1 State Key Laboratory of Robotics and System, Harbin Institute of Technology, Harbin 150080, China; 6120810528@hit.edu.cn (Y.F.); limt@hit.edu.cn (M.L.); 19s008087@stu.hit.edu.cn (S.Z.); 20S108240@stu.hit.edu.cn (C.W.); wguo01@hit.edu.cn (W.G.); hgcai@hit.edu.cn (H.C.); lnsun@hit.edu.cn (L.S.); wangpengfei@hit.edu.cn (P.W.); 2 Shaanxi Fast Auto Drive Group Co., LTD, Xi’an 710119, China; 00011683@fastgroup.cn; 3 Shenzhen Academy of Aerospace Technology, Shenzhen 518057, China

**Keywords:** spatial topological relation, convex hull, 6-intersection model

## Abstract

The spatial topological relations are the foundation of robot operation planning under unstructured and cluttered scenes. Defining complex relations and dealing with incomplete point clouds from the surface of objects are the most difficult challenge in the spatial topological relation analysis. In this paper, we presented the classification of spatial topological relations by dividing the intersection space into six parts. In order to improve accuracy and reduce computing time, convex hulls are utilized to represent the boundary of objects and the spatial topological relations can be determined by the category of points in point clouds. We verified our method on the datasets. The result demonstrated that we have great improvement comparing with the previous method.

## 1. Introduction

**Figure 1:**
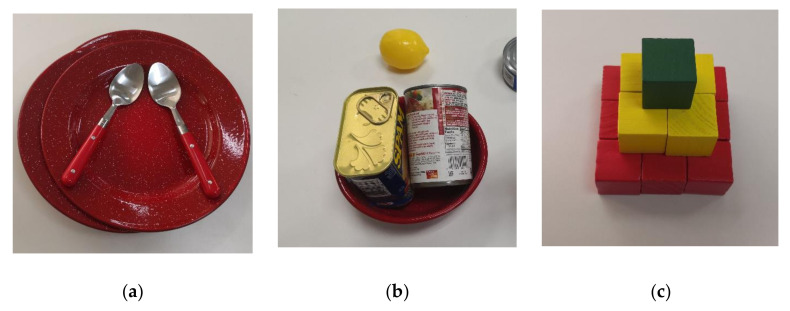
Multi-step operations for in cluttered scenes. (**a**) Move the plate with spoons on it; (**b**) Put a lemon into a full bowl; (**c**) Stack blocks like a tower.

To perform tasks autonomously under unstructured and cluttered scenes, a robot with artificial intelligence should have the ability to effectively perceive the complex spatial information and plan policy to complete tasks [1,2,3]. Planning a reasonable operation sequence by analyzing the spatial information may avoid fragile objects slipping or crushing [4,5,6,7]. For example, if we take out a dish from a pile of dishes with spoons on it, the spoons need to be removed beforehand (Figure 1a); if we want to put a lemon into a bowl, we should take out the cans first, as shown in Figure 1b; if we stack the blocks into a tower, we should make the base beforehand, as shown in Figure 1c. These are great challenges for the robot if the sequence of operations is unreasonable. However, most of the current robot tasks are limited to the operation of isolated objects on a plane [8], based on the template matching [9] or feature extraction methods [10]. In the scenes mentioned above, the operating space of robots is limited. The spatial relations between objects is complex, including physical contact, overlap, and occlusion [11]. Unsafe operations may cause the fragile objects falling on the ground, broken into pieces or other unexpected damages [12]. Therefore, it is necessary to analyze the spatial relations between cluttered objects and make an appropriate decision for the autonomous and safe operation of robotic manipulations [13].

Spatial topological relations are one of the important theories describing the relations between objects [14]. Spatial topological relations describe the adjacency and association relations about spatial points, lines, and surfaces [15]. A correct understanding of the spatial topological relations between objects is essential for the successful execution of robot actions [16]. The behavior decision of robots depends on the current state of the spatial topological relations [17]. On one hand, the spatial topological relations need to be accurately analyzed so that the robot performs the next operation correctly. On the other hand, taking work efficiency into consideration, the decision-making process cannot take too much time. Thus, the analysis of the spatial topological relation requires high accuracy and short computing time. Recently, a lot of works have come out to analyze the spatial topological relations in different ways [18,19,20,21], but it still has two challenges because of the cluttered scene: (1) the robot can only get partial point cloud on the surface of the object by vision sensors due to the occlusion; (2) the relations between objects are complex and difficult to be categorized; (3) the small deviation of the vision sensor may cause misclassification.

In this paper, we improved the classification method of spatial topological relation by dividing the cluttered space. Based on the distribution of point clouds in different spaces, the spatial topological relations can be defined, including cross, within, partial within, contain, partial contain, touch, and disjoint. The spatial topological relation can reasonably describe the relation between any two point clouds in the space. Meanwhile, the contour of partial point clouds was described by convex hulls, and the directed distance was utilized to determine the spatial relations of points. The main contributions in this work:

1. We simplified the widely used model of spatial topological relations and proposed the definition of particular formalism, which improved the accuracy of the spatial topological relation analysis in the cluttered scene.
2. We proposed the method that determines the spatial topological relation by the approximate expression of the object boundary and the spatial relations of points on cluttered objects. Deviation factor is employed to improve the robustness of the algorithm.

## 2. Related Works

In the past decades, researchers have done a lot of work about the spatial topological relations analysis [22,23,24]. The earliest research is used in geographic information systems (GIS). The focus of many research studies is on the formalism of spatial topological relations. The 9-intersection model (9IM) proposed by Egenhofer is one of the most widely used methods to represent spatial topological relations [25]. It is based on the point set topology theory to qualitatively describe the topological relations between targets. The 9IM defines the relations between objects as cross, touch, overlap, equal, within, contains, disjoint, and intersects by the information of the interior, boundary, and exterior of two objects. Based on the 9IM, Clementini [26] expanded the dimensions of relations, called dimensionally extended 9-intersection model (DE-9IM). Although the 9IM or DE-9IM can represent the spatial topological relations between objects, it requires the complete point, line, and surface information of the objects. However, the depth image obtained by robot vision often only contains part of the surface of the objects. Without the complete point clouds, the 9-intersection model cannot accurately represent the relations of objects.

Many research studies focus on the feature extraction of cluttered scenes. Nathan Silberman [27] proposed integer program formulation to infer the physical support relations by combining various methods, including geometric structure from depth, object attributes, and data-driven priors. Under the assumption of the Manhattan world, this method can infer simple support relations between objects in a complex indoor scene with cluttered and stacked objects. It ignores the overlapping situation, which might cause misclassification. On this basis, Panda [28] proposes the mapping inferring and linear programming method to expand the support relations between different entities in the scene, and inferred the relation types, such as “support from below”, “support from the side”, or “containment”. The support relations are expressed in a structure of tree, called support tree, and the support sequence of objects is obtained by performing on a traversal of reverse hierarchical sequence. This expression is reasonable for scene understanding and provides research foundation for robot operation planning. Kartmann [29] infers physically reasonable support relations between objects without any prior knowledge about the physical properties (mass distribution and friction coefficient). By the virtual force analysis, the uncertainty of the support relations is taken into account in the prediction. Jia [30] uses RGB-D data as input, performed a three-dimensional box on the surface of the object, extracted the bounding box representation features, and designed an energy function to determine the quality of the segmentation and the stability of the scene based on the support relations. This method represents and classifies objects for 3D scene understanding.

Some research utilized learning methods, such as support vector machines (SVMs) or artificial neural networks (ANNs), to infer the spatial topological relations. Rosman [31] used SVMs for the first time to describe the topology of two-dimensional spatial relations of objects, but their research is only applicable to simple objects without occlusion. Mojtahedzadeh [32] described a fast method to extract the support relations between pairs of objects in contact with each other by using the static balance principle. In addition to SVMs, they also use artificial neural networks (ANNs) and random forests to approximate the probability distribution of the relations between objects. However, this method only considers entities with convex polyhedral shapes (box, cylinder, and barrel), which limits its practical application. Zhuo [33] introduces an approach to infer support relations from a single image by Markov random field (MRF), integer linear programming, and SVMs framework.

To summarize, the 9IM or DE-9IM requires the complete surfaces, so it is not suitable for cluttered scenes. The methods based on geometric features pay attention to the nearby points, lines, and surface features of the object, but they ignore the overall features of objects. The methods based on learning methods use the generalization ability of ANNs or SVMs to infer the spatial relations between objects through the annotation of a large amount of data, but it ignores the physical features of object. Therefore, how to adjust the 9IM for cluttered scenes and propose the methods to solve it is the key to improving accuracy and adaptability of the spatial topological relation analysis.

## 3. Methods

Our model of spatial topological relations is based on the space division, including interior, boundary, and exterior, so that the spatial topological relations of any two three-dimensional objects can be described in detail by the intersection of point sets.

### 3.1. Definitions of Spatial Topological Relations

Let A be a point cloud obtained from depth camera. $A\subset {\mathbb{R}}^{3}$. The convex hull of point cloud  A is the smallest convex set that contains all the points of A. We present a formal definition of the boundary, interior, and exterior of A as follow:

**Definition** **1.** *The boundary of a point cloud*A*, denoted by*∂A*, is the convex hull of*A.

**Definition** **2.** *The interior of a point cloud*A*, denoted by*A°*, is the interior of*∂A*. There is no intersection between*A°*and*∂A*, so*A°∩∂A=∅.

**Definition** **3.** *The exterior of a point cloud*A*, denoted by*$A^{-}$*, is the exterior of*∂A. $A^{-}$*does not contain any point of*A*, so*$A\cap A^{-}=\emptyset$*. The three-dimensional space is completely divided into three parts*A°*,*∂A*, and*$A^{-}$*, i.e.,*$A{}^{\circ}\cup \partial A\cup A^{-}={\mathbb{R}}^{3}$.

**Definition** **4.** *The closure of a point cloud*A*is combining the boundary and the interior of the point cloud, denoted by*$A^{+}$*, i.e.,*$A^{+}=A{}^{\circ}\cup \partial A$.

Based on the definition of A°,  ∂A, $A^{-}$, and $A^{+}$, we have the following proposition:

**Proposition** **1.** $A\subset A^{+}$.

**Proof.**  Based on Definition 3, $A{}^{\circ}\cup \partial A\cup A^{-}={\mathbb{R}}^{3}$ and $A\cap A^{-}=\emptyset$. Due to Definition 4, $A^{+}=A{}^{\circ}\cup \partial A$, so we have $A^{+}\cup A^{-}={\mathbb{R}}^{3}$. Since $A\subset {\mathbb{R}}^{3}$, we have $A\subset A^{+}\cup A^{-}$. Together with $A\cap A^{-}=\emptyset$, it follows that $A\subset A^{+}$. □

The spatial topological relations between two point clouds, namely A and B, can be described as the relation between the A and B°, ∂B or B° and the relation between B and A°, ∂A or A°. There are:

(1) the parts of A located at the interior of ∂B, denoted by A∩B°;
(2) the parts of A located on ∂B $\left(A\cap \partial B\right)$;
(3) the parts of A located at the exterior of ∂B $\left(A\cap B^{-}\right)$;
(4) the parts of B located at the interior of ∂A $\left(A{}^{\circ}\cap B\right)$;
(5) the parts of B located on ∂A $\left(\partial A\cap B\right)$;
(6) the parts of B located at the exterior of ∂A $\left(A^{-}\cap B\right)$.

**Figure 2:**
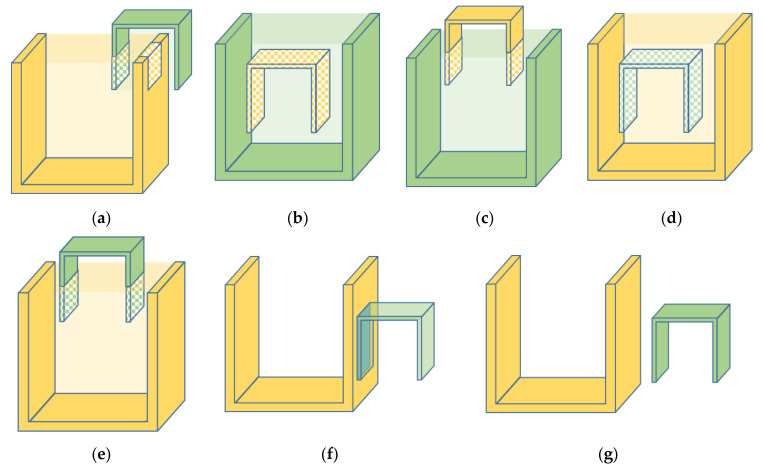
The spatial topological relations between two point clouds. The yellow area and the green area mean the region of point cloud A and B, respectively. The light yellow and light green areas represent the region of the convex hull of A and B, respectively. (**a**,**b**) are different spatial topological relations. (**a**) Cross; (**b**) Within; (**c**) Partial within; (**d**) Contain; (**e**) Partial contain; (**f**) Touch; (**g**) Disjoint.

Therefore, the spatial topological relation from A to B can be represented as a matrix $R\left(A,B\right)$:

(1) $$R\left(A,B\right)=\left(\begin{array}{ccc} A\cap B{}^{\circ} & A\cap \partial B & A\cap B^{-}\\ A{}^{\circ}\cap B & \partial A\cap B & A^{-}\cap B \end{array}\right),$$

which is called the 6-intersection model (6IM). The 6IM considers the intersection between point and boundary, ignoring the intersections between areas. So, the 6IM can be used in the scenes where only the point clouds on the surface of objects are available. Based on the 6IM, we define all the spatial topological relations from A to B, as shown in Figure 2. The yellow model is point cloud A and the green one is point cloud B. Taking into account all the circumstances, we have defined 7 spatial topological relations, i.e., cross, within, partial within, contain, partial contain, touch and disjoint. We give the definition of each spatial topological relation as follow.

**Definition** **4.** *If* A∩B° *and* A°∩B *are non-empty, then* A *crosses with B (Figure 2a).*

If A∩B°=¬∅, then we have $A^{+}\cap B{}^{\circ}=\neg \emptyset$ because of Proposition 1, which means that the closure of A has common part with the interior of B. Similarly, A°∩B=¬∅ means that the closure of B has common part with the interior of A. So, the closures of A and B have the common parts with the opposite interiors. In addition, if A∩B°=¬∅, then we have $A^{+}\cap \;B^{+}=\neg \emptyset$, which means the two point clouds have common closures. We define that A crosses with B if they have common closures and these closures intersect with the opposite interiors.

**Definition** **5.** *If* A∩B° *is non-empty,* $A\cap B^{-}$ *is empty and* A°∩B *is empty, then* A *is within B (Figure 2b).*

If A∩B°=¬∅, we have $A^{+}\cap B{}^{\circ}=\neg \emptyset$. It illustrates that the closure of point cloud A intersects with the interior of point cloud B. Based on Definition 3, if $A\cap B^{-}=\emptyset$, then we have $A\cap \left(B{}^{\circ}\cup \partial B\right)=\neg \emptyset$, which means that all the points of A are located in the boundary of B. A°∩B=∅ means that none of B’s points coincide with A’s interior. So, we define that A is within B by three conditions: (1) The closure of point cloud A intersects with the interior of point cloud B; (2) A and B have common interior, and all the points in A are located in the boundary of B; (3) None of B’s points coincides with A’s interior.

**Definition** **6.** *If* A∩B° *and* $A\cap B^{-}$ *are non-empty, and* A°∩B *is empty, then* A *is partial within B (Figure 2c).*

Similar with Definition 5, we give the definition of partial within by minor modifications. $A\cap B^{-}=\neg \emptyset$ means that some points from A are located outside of the boundary of B. So, we define that A is partial within B by three conditions: (1) The closure of point cloud A intersects with the interior of point cloud B; (2) A and B have common interior, and not all the points in A are located in the boundary of B; (3) None of B’s points coincide with A’s interior.

**Definition** **7.** *If* A∩B° *is empty,* A°∩B *is non-empty and* $A^{-}\cap B$ *is empty, then* A *contains B (Figure 2d).*

Contain is the reverse definition of within, and we define it by swapping the roles of *A* and *B* in Definition 5.

**Definition** **8.** *If* A∩B° *is empty,* A°∩B *is non-empty and* $A^{-}\cap B$ *is non-empty, then* A *partial contains B (Figure 2e).*

Similar with Definition 7, partial contain is the reverse definition of partial within.

**Definition** **9.** *If* A∩B° *and* A°∩B *are empty,* A∩∂B *and* ∂A∩B *are non-empty, then* A *touches B (Figure 2f).*

A∩B° and A°∩B are empty, which means that there is no intersection common area from the interiors of A and B. A∩∂B and ∂A∩B are non-empty, which means that the intersection between the boundaries of A and B is not empty. So, we define A touches B if the intersection between the boundaries of A and B is not empty and neither of the points of A or B is located in the opposite interior.

**Definition** **10.** *If* A∩B° *and* A°∩B *are empty, and at least one of* A∩∂B *and* ∂A∩B *is empty, then* A *is disjoint from B (Figure 2g).*

Different from Definition 9, at least one of A∩∂B and ∂A∩B is empty. So, if the intersection between the boundaries of A and B is not empty and the relation from A and B is not touch, we define it as disjoint.

We realize that the relations do not exist in some cases. To eliminate non-existent relations, we have the following proposition.

**Proposition** **2.** *If*A∩B°=∅*and*A∩∂B=∅*, then*$A\cap B^{-}=\neg \emptyset$.

**Proof.**  Due to Definition 3 and $A\cap {\mathbb{R}}^{3}=\neg \emptyset$, we have $A\cap \left(B{}^{\circ}\cup \partial B\cup B^{-}\right)=\neg \emptyset$, and then $\left(A\cap B{}^{\circ}\right)\cup \left(A\cap \partial B\right)\cup \left(A\cap B^{-}\right)=\neg \emptyset$. Because of A∩B°=∅ and A∩∂B=∅, so $A\cap B^{-}=\neg \emptyset$. □

The formal definition of the spatial topological relations is given by six different specifications with the values empty $\left(\emptyset \right)$, non-empty $\left(\neg \emptyset \right)$ or arbitrary $\left(*\right)$, shown in Table 1. Each relation expects disjoint is corresponded to a rule, and three situations are included in relation disjoint. Based on Proposition 2, we can distinguish non-existent relations, which are shown in Table 2. The relations are complete if summarizing them from Table 1 and Table 2.

### 3.2. Classification Criteria of Spatial Topological Relations

In order to infer the spatial topological relations by the 6IM, all the points in the point clouds of one object should be determined the relative position relations with the convex hull of another object. If a point locates in the convex hull of a point cloud, it must locate in the axis aligned-bounding box (AABB) of the point cloud.

**Proposition** **3.** *Given a point cloud*A*, let*$Box\left(B\right)$*an*∂B*be the axis aligned-bounding box and the convex hull of point cloud*B*. If*$A\cap Box\left(B\right)=\emptyset$*, then*A∩∂B=∅.

**Proof.**  The convex hull is the minimum convex bound of point cloud, and the axis aligned bounding box is convex bound either. So, we have $\partial B\subseteq Box\left(B\right)$. By $A\cap Box\left(B\right)=\emptyset$, we have A∩∂B=∅. □

It is easy and fast to evaluate whether a point is in AABB, in other words, whether the point is within the range of AABB at the three directions of coordinate axis, so we can speed up the classification of points. If a point is not in AABB, then the point is also not within the range of the convex hull. If a point is in AABB, the next step is to determine whether the point is in the convex hull. AABB is employed to represent the boundary of objects [16]. However, it is inappropriate for the spatial topological relation analysis. The reason is that AABB is an inexact approximation for the boundary of objects. The convex hull is the exact approximation for the boundary of objects and performs better than AABB.

**Figure 3:**
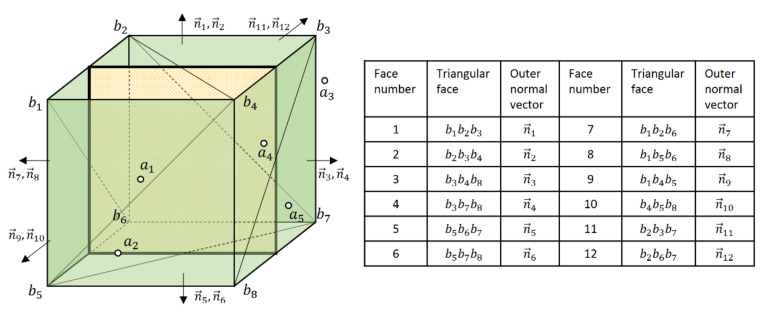
Some of points in A and the convex hull of B. The hollow dots represent points in A and the green cube represents the convex hull of B. Every three vertexes compose a triangular face of convex hull and it has an outer normal vertor ${\vec{n}}_{k}$, and k is the face number.

Take a cube convex hull for an example, as shown in Figure 3. Let $a_{i}\;\left(i=1,\ldots ,\;5\right)$ be the points of point cloud A, and the points $b_{j}\;\left(j=1,\ldots ,\;8\right)\;$ are the vertices of the convex hull of point cloud *B*. Every three points $b_{k1},\;b_{k2},\;b_{k3}\;\left(k=1,\ldots ,\;12\right)$ from $b_{j}$ constitute a triangular surface of the convex hull and ${\vec{n}}_{k}$ is an outer normal vector to the surface. So, as to determine the spatial topological relation between a point and convex hull, the next step is to iterate over the faces of the convex hull and to determine if the point is on the negative or positive side of the faces. The classical method from computation geometry is employed to determine if a point is inside a convex hull [34]. $a_{i}$ is inside the convex hull if ${\vec{n}}_{k}\cdot \left(a_{i}-b_{k1}\right)<0$ for all k, outside the convex hull if ${\vec{n}}_{k}\cdot \left(a_{i}-b_{k1}\right)>0$ for some of k, or on the boundary of the convex hull if ${\vec{n}}_{k}\cdot \left(a_{i}-b_{k1}\right)\leq 0$ for all k with equality occurring at least once.

**Figure 4:**
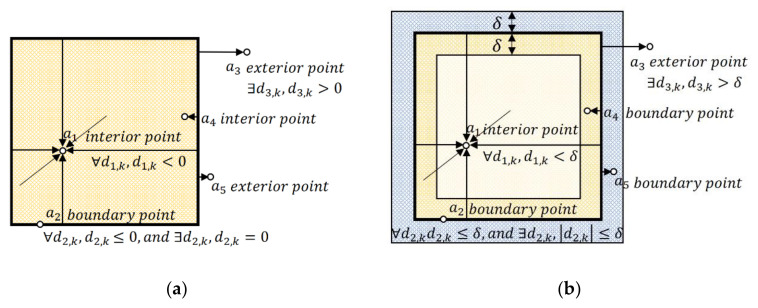
The classification method of point $a_{i}$. (**a**) Classical method; (**b**) Our method with the deviation factor δ. The directed distance $d_{i,\;k}$ is presented by the arrow from the face k of convex hull to the point $a_{i}$. If $d_{i,\;k}$ and ${\vec{n}}_{k}$ are in the same direction, then $d_{i,\;k}>0$, otherwise $d_{i,\;k}<0$.

Based on the distance formula, we define the directed distance from a point $a_{i}\;$ to a plane $b_{k1}b_{k2}b_{k3}$, that is:

(2) $$d_{i,\;k}=\frac{{\vec{n}}_{k}\cdot \left(a_{i}-b_{k1}\right)}{\left|{\vec{n}}_{k}\right|},$$

where ${\vec{n}}_{k}$ is an outer normal vector of the plane, and $\left|{\vec{n}}_{k}\right|>0$. $\left|d_{i,\;k}\right|$ means the distance from point $a_{i}$ to plane $b_{k1}b_{k2}b_{k3}$. By the definition of directed distance, the classical method is equal to:

(3) $$\left\{\begin{array}{cc} \forall k\in K,\;\;d_{i,\;k}<0, & a_{i}\;is\;interior\;point\\ \forall k\in K,\;\;d_{i,\;k}\leq 0\;and\;\exists k\in K,\;\;d_{i,\;k}=0, & a_{i}\;is\;boundary\;point\\ \exists k\in K,\;\;d_{i,\;k}>0, & a_{i}\;is\;exterior\;point \end{array}\right.,$$

where K is the set of the face number. In the example of cube convex hull, $K=\left\{k:k=1,\ldots ,\;12\right\}$. All the points of point cloud A can be classified by formula (3), as shown in Figure 4a. $a_{1}$ is an interior point because of $d_{1,\;k}<0$ for all k. $a_{2}$ is a boundary point on account of $d_{2,\;k}\leq 0$ for all the k, $d_{2,\;5}=0$ and $d_{2,\;6}=0$. $a_{3}$ is an exterior point by the reason of $d_{3,\;3}>0$ and $d_{3,\;4}>0$.

However, the position of $a_{i}$ obtained from the vision sensor may exist small deviation. In Figure 4a, $a_{4}$ and $a_{5}$ are supposed to be boundary point with small deviation. By formula (3), $a_{4}$ is determined as an interior point in view of $d_{4,\;k}<0$ for all the k, but $\left|d_{4,\;3}\right|$ and $\left|d_{4,\;4}\right|$ are quite small. Similarly, $a_{5}$ is misclassified as an exterior point due to $d_{5,\;3}>0$ and $d_{5,\;4}>0$, even if $\left|d_{5,\;3}\right|$ and $\left|d_{5,\;4}\right|$ are small. The reason of these misclassifications is that the determined condition of the boundary point in formula (3) is too strict and nearly unreachable under cluttered scenes. A small deviation of directed distance may cause misclassification, i.e., determining a boundary point to be an interior point or exterior point.

Based on the above descriptions, we relax the determined condition of boundary point by employing deviation factor to improve the robustness of our algorithm. By extending the upper and lower bounds of determine condition from $d_{i,\;k}=0$ to $\left|d_{i,\;k}\right|\leq \delta$, we have:

(4) $$\left\{\begin{array}{cc} \forall k\in K,\;\;d_{i,\;k}<-\delta , & a_{i}\;is\;interior\;point\\ \forall k\in K,\;\;d_{i,\;k}\leq \delta \;and\;\exists k\in K,\;\;\left|d_{i,\;k}\right|\leq \delta , & a_{i}\;is\;boundary\;point\\ \exists k\in K,\;\;d_{i,\;k}>\delta , & a_{i}\;is\;exterior\;point \end{array}\right.,$$

where δ is the deviation factor. Generally, δ is a small positive value, and the larger it takes, the more boundary points will be determined. So, the value of δ is usually equal to the deviation of point clouds. By formula (3), points $a_{n}$ from point cloud A are classified, as shown in Figure 4b. The classifications of $a_{1},$ $a_{2}$, and $a_{3}$ are the same as the classical method. Unlike the classical method, $a_{4}$ and $a_{5}$ are determined as boundary points as they are supposed to be by our method.

If the point $a_{i}$ is an interior point for the convex hull of B, then A∩B°=¬∅. If the point p is a boundary point, then A∩∂B=¬∅. Otherwise, if the point p is an exterior point, then $A\cap B^{-}=\neg \emptyset$. We traverse the points in point cloud A unless A∩B°, A∩∂B, and $A\cap B^{-}$ are all non-empty. We stop the loop of point cloud A when A∩B°, A∩∂B, and $A\cap B^{-}$ are all non-empty, because the rest of calculation will not change the results. By this way, the spatial topological relations can be decided by the 6IM. The whole approach is described in Algorithm 1.
**Algorithm 1.** Spatial Topological Relation Analysis Algorithm. **Input**: 3D point cloud of each object **Output**: The spatial topological relations between the objects **Initialize:** Create convex hull and AABB of each object from point cloud **begin** **for** each object A **do** **for** each object B **
do** **for** each point $a_{i}\;$ in object A **do** **if**
$a_{i}$ is not in $Box\left(B\right)$ **then**
A∩B°=∅, A∩∂B=∅ and $A\cap B^{-}=\neg \emptyset$
**continue** Compute the relative position of p by formula (4)      Check A∩B°, A∩∂B and $A\cap B^{-}$ for loop termination **end** **end** **end** **for** each object A **do** **for** each object B **do** **if**
A∩B°=¬∅ and A°∩B=¬∅
**then**
$R\left(A\to B\right)=cross$ **else if**
A°∩B=∅ **if**
$A\cap B^{-}=\emptyset$
**then**
$R\left(A\to B\right)=within$ **else**
$R\left(A\to B\right)=\mathrm{partial}\;\mathrm{within}$ **else if**
A∩B°=∅ **if**
$A^{-}\cap B=\emptyset$
**then**
$R\left(A\to B\right)=contain$ **else**
$R\left(A\to B\right)=partial\;contain$ **else if**
A∩B°=∅, A∩∂B=¬∅, A°∩B=∅ and ∂A∩B=¬∅      **then**
$R\left(A\to B\right)=touch$ **else**
$R\left(A\to B\right)=disjoint$**end****end****end**

## 4. Experimental Results

To verify the accuracy and the rapidity of our spatial topological relation analysis method described in Section 3, we have done a series of experiments on the point clouds generated from the International Institute of information Technology (IIIT) RGBD dataset and the Yale-CMU-Berkeley (YCB) benchmarks.

### 4.1. Pretreatment

#### 4.1.1. IIIT RGBD Dataset

The IIIT RGBD dataset contains seven scenes with different types of physical interactions between objects, such as supporting from below, supporting from the side and inclusion [35]. Due to the occlusion by each other, all the RGBD images are part of the point clouds representing the surface of objects. Each RGBD image is segmented by semantic annotation. We reconstructed point clouds of objects from RGBD images by the point cloud registration method [36]. Additionally, the convex hull of each point cloud was obtained by the Quickhull method [37].

#### 4.1.2. YCB Benchmarks

The YCB benchmarks are designed for robot manipulation. The model set contains different kinds of objects, such as food, tools, and kitchen items [38]. Each object has the corresponding 3D model reconstructed from the merged point clouds with high precision. We chose several objects, made 7 scenes, and removed outliers by Point Cloud Library (PCL).

### 4.2. Results

**Figure 5:**
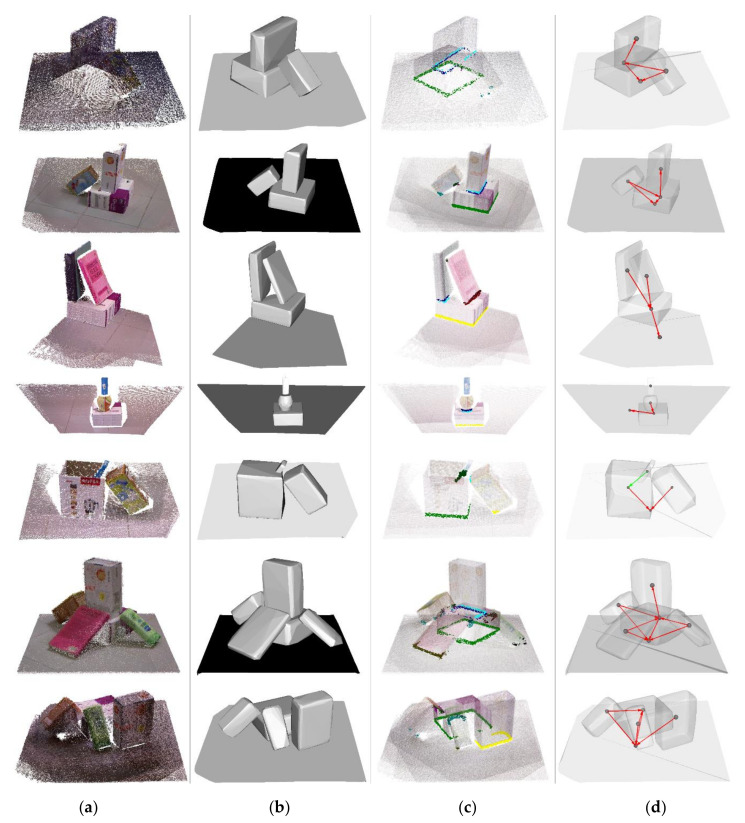
Visualized results of our spatial topological relation analysis on the IIIT RGBD dataset: (**a**) The original images of cluttered scenes 1 to 7; (**b**) 3D models of convex hull; (**c**) Boundary points and interior points; (**d**) Spatial topological relations between objects.

The reconstruction point clouds of the IIIT RGBD dataset are shown in Figure 5a. The point cloud of ground has little effect on the result so only the point cloud near the objects is reserved. Then, we use filter to remove outlier points of point clouds. Due to the semantic annotation, all the objects can be reconstructed respectively. In this way, all the objects are separated from each other. We display all objects, including the ground, in Figure 5a. Scene 1 and scene 2 are similar. One box lays on the ground, with a box leaning on it and another box putting upright on it. In scene 3, one box lays on the ground and two books putting on it. Because the images are taken near the corner of the walls, photos cannot be taken in some perspective and the images are insufficient for complete 3D reconstruction. As a result, the point clouds of box and books are incomplete. In scene 4, a box lays on the ground, and a hollow jar is placed on it. In the hollow jar, a rod-like object inserts inside it. Due to the insufficient RGBD images in the IIIT RGBD dataset, only a small part of objects can be reconstructed. The 3D reconstruction of scene 4 shows that the rod-like object seems to levitate in the air. A large area of point cloud missing may cause failure. In scene 5, a solid box leans on a hollow box, and there is a bar placed in the hollow box. Different from scene 4, we have plenty of RGBD images taken from multiple perspectives of scene 5. So, the point clouds of objects in scene 5 are relatively complete, compared with scene 4. Scene 6 is quite cluster, with five objects crowded in the limited space. A box is placed horizontally on the ground, and three boxes lean on it and one box is placed vertically on it. Scene 7 is similar to scene 6, and there are four objects in this scene. One box is placed horizontally on the ground, and two boxes lean on it. Besides, a box is placed isolated on the ground. Although scene 6 and scene 7 are cluster and objects cover each other, due to sufficient images from all perspectives, we can reconstruct most of the point clouds from the surface of objects.

Because all the RGBD images are taken by the depth camera, so only the surface of point clouds is captured. Although the point cloud of each object can be obtained separately, the 3D reconstruction is fragmentary and only contains surface point clouds. The convex hull of point clouds is shown in Figure 5b. Due to the incomplete point clouds, the convex hulls are the subsets of the actual convex hulls of point clouds.

Despite these obstacles above-mentioned existing, our method can still analyze the spatial location of points. By the method in Section 3.2, the boundary points and interior points of each point cloud were classified, as shown in Figure 5c. The boundary points and interior points between different objects are drawn in different colors. By the definitions in Section 3.1, we can decide the spatial topological relations between objects, as shown in Figure 5d. The red line represents touch, and the direction of arrows represents the direction of relation as A→B. The green line represents partial contain. The relation disjoint is so common that we ignore its visualization. In scene 1 and 2, the results show that the box laying on the ground touches ground and all the other boxes. In scene 3, the two books touch each other and touch the box at the same time. In scene 4, the box touches the hollow jar and the ground simultaneously. However, due to the lack of point cloud, only a small part of the hollow jar can be reconstructed. As a result, the point cloud of the rod-like object is far away from the hollow jar, and the spatial topological relation between them, which is supposed to be partial within, have been misjudged as disjoint. In scene 5, the hollow box touches the ground and partial contains the blue bar. The solid box touches the ground, but the relation between the hollow box and the solid box is misjudged as disjoint, which should be touch. The reason is that the point cloud is so sparse at the contacting surface that few points of the solid box are located in the convex hull the hollow box. Despite the scenes are quite clustered and messy, our method performed well in scene 6 and 7. All the relations obtained by our method are identical to the ground truth. In scene 6, the lying box touches all the other boxes. In scene 7, the isolated box is disjoint with the other boxes.

We have compared our method with the feature extraction method, learning method, and AABB method on the accuracy and the computing time. The accuracy means the number of the relations which are correctly classified divided by the number of all the relations in one scene. The criterion for determining whether the classification of spatial topological relations is correct is to compare them with the ground truth from the dataset. In the feature extraction method [35], the definition of spatial topological relations is different from ours, so we combine “support from below” and “support from the side” to touch. If the relation is “containment”, we determine it is correct no matter whether the ground truth is partial contain or contain. By the learning method [31], we got contact point networks of point cloud by SVMs and classified relations by k-means method. The AABB method is using AABB, instead of convex hull, to represent the boundary of objects, and the other is same as our method. The computing time is the time of the analysis of all the relations in one scene. The results are shown in Table 3. In every scene, our method performed obviously better than the feature extraction method [35] on the accuracy and the computing time. There are 55 relations in 7 different scenes. Our method has correctly analyzed 53 relations and cost 131.8 s in total. As a comparison, the feature extraction method, the learning method, and the AABB method have correctly analyzed 41, 40, and 36 relations, with costing 1478.2, 231.1, and 17.7 s, respectively. The average accuracy of our method on the IIIT RGBD dataset is 96.4%, which is 21.9% higher than 74.5% accuracy of the feature extraction method, 23.7% higher than 72.7% accuracy of the feature extraction method, and 30.9% higher than 65.5% accuracy of the AABB method. In addition, the average time of our method is 2.4 s, which is faster than 26.9 s by the feature extraction method and 4.2 s by the learning method. Although our method is slower than the AABB method, the accuracy of our method is much higher.

**Figure 6:**
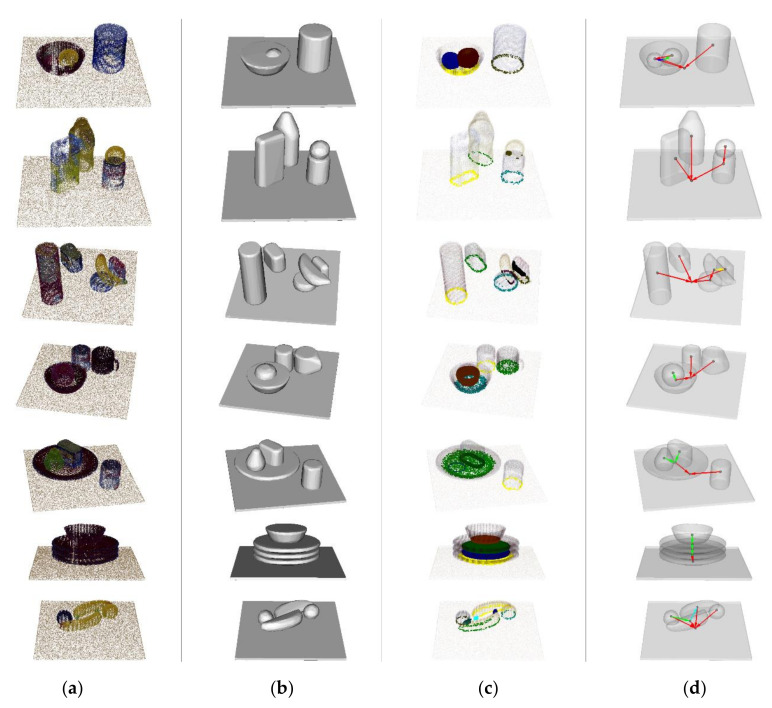
Visualized results of our spatial topological relation analysis on the YCB benchmarks: (**a**) The original images of cluttered scenes 8 to 14; (**b**) 3D models of convex hull; (**c**) Boundary points and interior points; (**d**) Spatial topological relations between objects.

Different from the IIIT RGBD dataset, the YCB benchmarks provide dense and high-resolution point clouds of objects. All the 3D point clouds are reconstructed by precise stitching, as shown in Figure 6a. The image of scene 8 shows that there are a strawberry and a lemon in the bowl placed on the table, and a master chef can is far from them. In scene 9, a mustard bottle, a sugar box, and a tomato soup can are placed on the table, and there is a lemon on the tomato soup can. Scene 10 is clustered. In scene 10, a tuna fish can and a gelatin box are very close, but they are not touching with each other, and a banana is placed on the tuna fish can. Besides, a chips can and a potted meat can are living away from other objects. In scene 11, a mug, a bowl, and a tomato soup can are placed closely on the table, and the bowl contains an orange. In scene 12, there is a plate on the table, with a bear and potted meat can on it, and a tomato soup can is isolated. Scene 13 is a typical case in the kitchen. A stack of plates is placed on the table, and a bowl is placed on the top of them. In scene 14, two bananas are placed mostly parallel on the table, with a plum and a lemon aside. All the point clouds of objects in each scene are dense, with few noise points in them. This is beneficial to the spatial topological relation analysis. Another advantage is that the YCB benchmarks contain a variety of relations and this is suitable for the verification of our method.

The convex hull of point clouds is shown in Figure 6b. Due to the density and the number of points of the point clouds in the YCB benchmarks are much larger than these in the IIIT RGBD dataset, the completion of convex hulls in the YCB benchmarks are better. The boundary points and interior points of each point cloud are classified, as shown in Figure 6c. We found that the number of boundary points and interior points are much larger than the IIIT RGBD dataset. The reason is that the spatial topological relations are plentiful in the YCB benchmarks. The spatial topological relations between objects are shown in Figure 6d. The red line represents touch, and the direction of arrows represents the direction of relation as A→B. The cyan, green, blue, magenta, and yellow line represents cross, within, partial within, contain, and partial contain, respectively. In scene 8, our result shows that the strawberry is within the bowl and the lemon is partial within the bowl, which is consistent with the ground truth. In scene 9, the mustard bottle, tomato soup can, and sugar box touches the table, and the lemon touches the tomato soup can. Scene 10 is a special case. Most of relations, except the relation between the banana and the gelatin box, are classified correctly. The misclassification is mainly caused by the common sense of human beings. The banana is not a container, so the relation generally cannot be considered as contain. However, based on the definition of 6IM, the relation is determined as partial contain. In scene 11, the orange is partial within the bowl, and other objects touch the table. Scene 12 is similar to scene 11 where the potted meat can and the bear are partial within the plate, which touches the table together with the tomato soup can. In scene 13, the plate at the bottom of stack touches the table and other objects are partial within the object below from top to bottom. In scene 14, the relation between two bananas is complex so it is determined as cross. Because of the same reason as scene 10, the relation between the plum and the banana, which is supposed to be touch, is misidentified as partial within.

We have also compared our method with the feature extraction method on the accuracy and the computing time, as shown in Table 4. Same as the results on the IIIT RGBD dataset, our method is significantly better than the other methods in terms of accuracy and calculation time. There are 75 relationships in 7 different scenes. Our method has correctly analyzed 71 relationships and took 131.7 s in total. As a comparison, the feature extraction method, the learning method, and the AABB method have correctly analyzed 64, 59, and 57 relationships, which took a total of 3002.4, 175.6, and 10.9 s. The number of relations and the points of each objects in YCB benchmarks are larger than these in the IIIT RGBD dataset, so our method and the feature extraction method took much more time on the calculation of spatial topological relations. The average accuracy of our method on the IIIT RGBD dataset is 94.7%, which is 9.3% higher than the 85.3% accuracy of the feature extraction method, 16.0% higher than the 78.7% accuracy of the AABB method, and 18.7% higher than the 76.0% accuracy of the AABB method. In addition, the average time of our method is 1.8 s, which is significantly faster than 40.0 s of the feature extraction method and 23.4 s of the learning method. Although the AABB method is really fast, the accuracy of it is the worst among these methods.

## 5. Discussion

The results confirmed that our method had advantages over the other methods on the accuracy. The reason is that our method effectively classifies the relations between objects and use the overall features of the point cloud to analyze the spatial topological relations. Currently, the spatial topological relations are mainly defined by the intersections of points, lines, and regions. However, in the cluttered scenes, the point clouds of objects are incomplete and the shapes of them are unpredictable. Meanwhile, only the point clouds on the surface of objects can be perceived by vision sensors. Without obvious region features, the current spatial topological relation methods cannot work well. Our method used convex hull to represent the boundary of objects. Since convex hulls contain the boundary feathers of point clouds, our method improved the accuracy and saves computing time for the interaction analysis between point clouds. In addition, we proposed the deviation factor to improve the robustness of our method. Although our method based on convex hulls has done a certain degree of region interpolation, however, it is not suitable for the scenes with point clouds severely missing. The methods based on object stability inferring may be helpful for further improvement on the accuracy in these extreme scenes.

## 6. Conclusions

In summary, we have identified 6IM to describe the spatial topological relations in cluttered scenes and its classification by calculating the relations between points and convex hulls. Different from others, our method takes the convex hulls as the approximate expression of the boundary of objects. Due to the reasonable definition and calculation process, our method is suitable for the cluttered scenes with partial, hollow, and complex point clouds. The rapidity and the accuracy of our method are verified on the IIIT dataset and the YCB benchmarks, on which we have improvement in every scene comparing with other methods.

In the future, we will improve the accuracy of the spatial topological relation analysis in the scenes with point clouds severely missing by stability inferring. Based on the spatial topological relation analysis, we will design the robot grasping strategy to realize automatic object sorting in cluttered scenes.

## Figures and Tables

**Table 1:** The definition of the spatial topological relations between two point clouds.

$\mathrm{Relation}\left(A\to B\right)$	A∩B°	A∩∂B	$A\cap B^{-}$	A°∩B	∂A∩B	$A^{-}\cap B$
cross	¬∅	*	*	¬∅	*	*
within	¬∅	*	∅	∅	*	*
partial within	¬∅	*	¬∅	∅	*	*
contain	∅	*	*	¬∅	*	∅
partial contain	∅	*	*	¬∅	*	¬∅
touch	∅	¬∅	*	∅	¬∅	*
disjoint	∅	¬∅	*	∅	∅	¬∅
	∅	∅	¬∅	∅	¬∅	*
	∅	∅	¬∅	∅	∅	¬∅

**Table 2:** Non-existent relations.

A∩B°	A∩∂B	$A\cap B^{-}$	A°∩B	∂A∩B	$A^{-}\cap B$
∅	¬∅	*	∅	∅	∅
∅	∅	∅	∅	¬∅	*
∅	∅	∅	∅	∅	∅

**Table 3:** Accuracy (%) and time (s) on the IIIT RGBD Dataset.

Scene	Our Method		Feature Extraction		Learning Method		AABB Method	
	Accuracy	Time	Accuracy	Time	Accuracy	Time	Accuracy	Time
1	100	10.2	66.7	41.8	83.3	25.7	50.0	1.4
2	100	15.8	83.3	197.9	83.3	44.3	66.7	3.0
3	100	27.5	66.7	116.0	100	33.8	83.3	2.1
4	83.3	3.1	83.3	12.8	83.3	7.9	66.7	0.9
5	83.3	3.5	66.7	30.1	66.7	13.9	66.7	1.4
6	100	55.3	86.7	775.6	60.0	77.1	60.0	4.9
7	100	16.4	60.0	304.0	60.0	28.4	70.0	4.0

**Table 4:** Accuracy (%) and time (s) on the YCB benchmarks.

Scene	Our Method		Feature Extraction		Learning Method		AABB Method	
	Accuracy	Time	Accuracy	Time	Accuracy	Time	Accuracy	Time
8	100.0	14.6	90.0	365.3	80.0	12.0	80.0	1.6
9	100.0	13.0	100.0	176.0	90.0	12.8	90.0	1.2
10	93.3	19.4	93.3	341.8	86.7	22.3	80.0	2.0
11	100.0	20.1	90.0	523.2	70.0	24.1	50.0	1.2
12	90.0	14.2	70.0	305.2	80.0	16.5	80.0	1.0
13	100.0	42.6	70.0	1033.9	70.0	78.1	70.0	3.2
14	80.0	7.8	80.0	257.0	70.0	9.8	80.0	0.7

## References

[1] Bing Z., Meschede C., Röhrbein F., Huang K., Knoll A.C. (2018). A survey of robotics control based on learning-inspired spiking neural networks. Front. Neurorobotics.

[2] Bing Z., Meschede C., Chen G., Knoll A., Huang K. (2020). Indirect and direct training of spiking neural networks for end-to-end control of a lane-keeping vehicle. Neural Netw..

[3] Bing Z., Lemke C., Cheng L., Huang K., Knoll A. (2020). Energy-efficient and damage-recovery slithering gait design for a snake-like robot based on reinforcement learning and inverse reinforcement learning. Neural Netw..

[4] Danielczuk M., Kurenkov A., Balakrishna A., Matl M., Wang D., Martín-Martín R., Garg A., Savarese S., Goldberg K. Mechanical search: Multi-step retrieval of a target object occluded by clutter. Proceedings of the 2019 International Conference on Robotics and Automation (ICRA).

[5] Imran A., Kim S.-H., Park Y.-B., Suh I.H., Yi B.-J. (2019). Singulation of Objects in Cluttered Environment Using Dynamic Estimation of Physical Properties. Appl. Sci..

[6] Murali A., Mousavian A., Eppner C., Paxton C., Fox D. 6-dof grasping for target-driven object manipulation in clutter. Proceedings of the 2020 IEEE International Conference on Robotics and Automation (ICRA).

[7] Jo H., Song J.-B. (2020). Object-Independent Grasping in Heavy Clutter. Appl. Sci..

[8] Hang K., Morgan A.S., Dollar A.M. (2019). Pre-grasp sliding manipulation of thin objects using soft, compliant, or underactuated hands. IEEE Robot. Autom. Lett..

[9] Shafii N., Kasaei S.H., Lopes L.S. Learning to grasp familiar objects using object view recognition and template matching. Proceedings of the 2016 IEEE/RSJ International Conference on Intelligent Robots and Systems (IROS).

[10] Spiers A.J., Liarokapis M.V., Calli B., Dollar A.M. (2016). Single-grasp object classification and feature extraction with simple robot hands and tactile sensors. IEEE Trans. Haptics.

[11] Naseer M., Khan S., Porikli F. (2018). Indoor scene understanding in 2.5/3d for autonomous agents: A survey. IEEE Access.

[12] Zheng B., Zhao Y., Yu J., Ikeuchi K., Zhu S.-C. (2015). Scene understanding by reasoning stability and safety. Int. J. Comput. Vis..

[13] Battaglia P.W., Hamrick J.B., Tenenbaum J.B. (2013). Simulation as an engine of physical scene understanding. Proc. Natl. Acad. Sci. USA.

[14] Zlatanova S., Rahman A.A., Shi W. (2004). Topological models and frameworks for 3D spatial objects. Comput. Geosci..

[15] Theobald D.M. (2001). Topology revisited: Representing spatial relations. Int. J. Geogr. Inf. Sci..

[16] Ziaeetabar F., Aksoy E.E., Wörgötter F., Tamosiunaite M. Semantic analysis of manipulation actions using spatial relations. Proceedings of the 2017 IEEE International Conference on Robotics and Automation (ICRA).

[17] Aksoy E.E., Aein M.J., Tamosiunaite M., Wörgötter F. Semantic parsing of human manipulation activities using on-line learned models for robot imitation. Proceedings of the 2015 IEEE/RSJ International Conference on Intelligent Robots and Systems (IROS).

[18] Shen J., Zhang L., Chen M. (2018). Topological relations between spherical spatial regions with holes. Int. J. Digit. Earth.

[19] Sjöö K., Aydemir A., Jensfelt P. (2012). Topological spatial relations for active visual search. Robot. Auton. Syst..

[20] Long Z., Li S. (2013). A complete classification of spatial relations using the Voronoi-based nine-intersection model. Int. J. Geogr. Inf. Sci..

[21] Shen J., Zhou T., Chen M. (2017). A 27-intersection model for representing detailed topological relations between spatial objects in two-dimensional space. ISPRS Int. J. Geo-Inf..

[22] Fu L., Yin P., Li G., Shi Z., Liu Y., Zhang J. (2018). Characteristics and Classification of Topological Spatial Relations in 3-D Cadasters. Information.

[23] Xu J., Cao Y., Zhang Z., Hu H. Spatial-temporal relation networks for multi-object tracking. Proceedings of the 2019 IEEE International Conference on Computer Vision (ICCV).

[24] Zhou M., Guan Q. (2019). A 25-Intersection Model for Representing Topological Relations between Simple Spatial Objects in 3-D Space. ISPRS Int. J. Geo-Inf..

[25] Egenhofer M.J., Franzosa R.D. (1991). Point-set topological spatial relations. Int. J. Geogr. Inf. Syst..

[26] Clementini E., Di Felice P., Van Oosterom P. A small set of formal topological relationships suitable for end-user interaction. Proceedings of the 1993 International Symposium on Spatial Databases (ISSD).

[27] Silberman N., Hoiem D., Kohli P., Fergus R. Indoor segmentation and support inference from rgbd images. Proceedings of the 2012 European conference on computer vision (ECCV).

[28] Panda S., Hafez A.A., Jawahar C. Learning support order for manipulation in clutter. Proceedings of the 2013 IEEE/RSJ International Conference on Intelligent Robots and Systems (IROS).

[29] Kartmann R., Paus F., Grotz M., Asfour T. (2018). Extraction of physically plausible support relations to predict and validate manipulation action effects. IEEE Robot. Autom. Lett..

[30] Jia Z., Gallagher A.C., Saxena A., Chen T. (2014). 3d reasoning from blocks to stability. IEEE Trans. Pattern Anal. Mach. Intell..

[31] Rosman B., Ramamoorthy S. (2011). Learning spatial relationships between objects. Int. J. Robot. Res..

[32] Mojtahedzadeh R., Bouguerra A., Schaffernicht E., Lilienthal A.J. (2015). Support relation analysis and decision making for safe robotic manipulation tasks. Robot. Autonom. Syst..

[33] Zhuo W., Salzmann M., He X., Liu M. Indoor scene parsing with instance segmentation, semantic labeling and support relationship inference. Proceedings of the 2017 IEEE Conference on Computer Vision and Pattern Recognition (CVPR).

[34] Schneider P., Eberly D.H. (2002). Geometric Tools for Computer Graphics.

[35] Panda S., Hafez A.A., Jawahar C. (2016). Single and multiple view support order prediction in clutter for manipulation. J. Intell. Robot. Syst..

[36] Zha F., Fu Y., Wang P., Guo W., Li M., Wang X., Cai H. (2020). Semantic 3D Reconstruction for Robotic Manipulators with an Eye-In-Hand Vision System. Appl. Sci..

[37] Barber C.B., Dobkin D.P., Huhdanpaa H. (1996). The quickhull algorithm for convex hulls. ACM Trans. Math. Softw. (TOMS).

[38] Calli B., Singh A., Bruce J., Walsman A., Konolige K., Srinivasa S., Abbeel P., Dollar A.M. (2017). Yale-CMU-Berkeley dataset for robotic manipulation research. Int. J. Robot. Res..

